# Modifications of liver stiffness and CXCL4, TGF-β1 and HGF are similar in HCV- and HIV/HCV-infected patients after DAAs

**DOI:** 10.1038/s41598-021-89370-6

**Published:** 2021-05-10

**Authors:** Mercedes Márquez-Coello, Ana Arizcorreta, María Rodríguez-Pardo, Francisco Illanes-Álvarez, Denisse Márquez, Sara Cuesta-Sancho, José-Antonio Girón-González

**Affiliations:** 1 Servicio de Medicina Interna y Enfermedades Infecciosas, Facultad de Medicina, Hospital Universitario Puerta del Mar, Instituto para la Investigación e Innovación en Ciencias Biomédicas de Cádiz (INiBICA), Universidad de Cádiz, Avda Ana de Viya s/n, 11009 Cádiz, Spain; 2 Servicio de Aparato Digestivo, Hospital Universitario Puerta del Mar, Instituto para la Investigación e Innovación en Ciencias Biomédicas de Cádiz (INiBICA), Cádiz, Spain

**Keywords:** Virus-host interactions, Diagnostic markers, HIV infections, Hepatitis C, Liver fibrosis

## Abstract

The objective of this work was to identify predictive factors of fibrosis regression after direct antiviral agents (DAAs) in HCV-monoinfected and HIV/HCV-coinfected patients. This was a prospective study of HCV-monoinfected (n = 20), HIV/HCV-co-infected (n = 66) patients and healthy controls (n = 15). Patients had started DAAs and achieved sustained virological response. Liver stiffness (LS) and serum concentrations of profibrotic transforming growth factor (TGF)-β1 and CXC chemokine ligand 4 (CXCL4) and antifibrotic HGF hepatocyte growth factor (HGF) were analyzed at baseline (M0) and 12 months after starting DAAs (M12). A M12 LS achievement of ≤ 9.5 kPa was considered the cutoff point to discharge from a liver clinic. The LS decrease from M0 to M12 was 34%. No significant differences were observed in LS decline between HCV- and HIV/HCV-infected individuals. Changes of serum CXCL4, TGF-β1 and HGF levels did not correlate with LS improvement. 16 out from 56 patients (28%) with a baseline LS > 9.5 achieved a M12 LS ≤ 9.5. HCV-monoinfected and HIV/HCV coinfected patients experienced a significant reduction of LS after sustained virological response. This improvement did not correlate with changes in serum profibrotic or antifibrotic markers. A 29% of those with a baseline LS > 9.5 achieved a LS under this cutoff point.

## Introduction

The achievement of a sustained virological response (SVR) after treating chronic hepatitis C virus (HCV) infection reduces all-cause mortality and liver complications, such as cirrhosis, liver decompensation, and hepatocellular carcinoma^1^. The impact is even more relevant in human immunodeficiency virus (HIV)-coinfected patients, in which HCV-induced disease progresses more rapidly than in monoinfected patients^2^.

It has been appreciated that inflammatory and coagulation pathways are both intrinsically involved in the pathophysiology of liver damage^3^. Blood platelets are activated in inflammatory and immune processes and in the hemostatic disorders in the liver, releasing active compounds [such as transforming growth factor (TGF)-β1 and CXC chemokine ligand 4 (CXCL4)]^4^. These proteins stimulate the fibrinogenesis and mitogenesis of Ito cells in the liver^5^. Both TGF-β1 and CXCL4 are circulating biomarkers of tissue fibrosis^6,7^.

Contradictory results have been published about serum concentrations of these markers. Some articles have demonstrated an increase of serum levels of TGF-β1 in HCV-monoinfected patients over time, mainly in patients with progressive liver fibrosis^8,9^. However, other authors have observed that liver stiffness values are inversely correlated with TGF-β1^10^, mainly in HIV/HCV coinfected patients^11,12^. Anti-HCV therapy with interferon alpha and ribavirin affects the tissue expression of TGF-β1, and may thereby modulate hepatic fibrogenetic events^13^.

In the case of CXCL4, serum levels are significantly increased in HCV‐infected patients with moderate to severe liver fibrosis compared to individuals with no or only mild fibrosis or to healthy controls^4,7^. However, individuals with severe fibrosis and cirrhosis experience a decrease in CXCL4 serum levels owing to reduced numbers of platelets^4^.

In contrast with these profibrogenic molecules, hepatocyte growth factor (HGF) is a powerful inhibitor of hepatic stellate cells activation^14^. In addition to a potential antifibrogenic effect, several studies have shown that HGF is involved in hepatic cell regeneration^15^ and has a proangiogenic role in chronic viral liver disease^16^. Another process wherein HGF has been involved is the development of hepatocellular carcinoma (HCC)^17^. Our group has demonstrated that high serum HGF levels are associated with greater liver fibrosis in HCV-infected patients^18^. Serum HGF concentrations did not decrease after response to interferon plus ribavirin^19^.

Serum-based fibrosis markers, like AST to platelets ratio index (APRI)^20^ and Fibrosis-4 (FIB-4) score^21^, are readily available, but their diagnostic performance is suboptimal in both low- and high prevalence scenarios, with high false positive rates for detection of cirrhosis^22^. Transient elastometry (TE) is a noninvasive procedure that assesses liver fibrosis by measuring liver stiffness (LS). LS measurement has shown a high accuracy for detecting liver fibrosis in HCV-infected patients, either with^21^ or without HIV-coinfection^22^. TE has superior diagnostic performance in identifying cirrhosis, with lower rates of misclassification of patients^20^. Several studies, carried out mostly among monoinfected individuals, have reported a significant decrease in liver stiffness after treatment^20,21^. Nevertheless, there is scarce data regarding the impact of SVR on liver stiffness within HIV/HCV patients treated with DAAs^23–28^. Furthermore, data about the relationship between modifications of liver fibrosis, both in HCV-monoinfected and HIV/HCV coinfected patients, and the serum concentrations of serum markers of fibrosis (CXCL4 or TGF-β1) or regeneration/proliferation (HGF), are also scarce and controversial^9,11,12^, even though these parameters could be markers of fibrosis evolution.

Our objectives were: (1) analyze the liver stiffness improvement, measured by transient elastography, in patients with SVR after DAAs, and those factors related with it. (2) Compare the liver stiffnes evolution in HIV/HCV coinfected and HCV-monoinfected patients. (3) Correlate changes of serum markers of fibrosis (CXCL4 or TGF-β1) or proliferation (HGF), and modification of LS.

## Results

### Baseline results

A total of 86 patients naïve to DAA underwent HCV, achieving SVR, and had two TE and serum samples separated by 12 months. Baseline characteristics of the healthy controls, HCV-monoinfected and HIV/HCV coinfected patients are shown in Table 1.

**Table 1:** Baseline characteristics of the population of the cohort.

Characteristic	Healthy controls (n = 15) (A)	HCV-infected patients			*p*			
		Global (n = 86) (B)	HCV monoinfected patients (n = 20) (C)	HIV/HCV coinfected patients (n = 66) (D)	A versus B	A versus C	A versus D	C versus D
Male sex [n (%)]	10 (67)	63 (73)	8 (35)	58 (88)	0.831	0.222	0.103	< 0.001
Age (years), median (IQR)	55 (48–61)	55 (51–59)	60 (56–67)	54 (50–56)	0.230	0.195	0.299	< 0.001
PWID		35 (41)	1 (5)	34 (52)				< 0.001
**HCV genotype [n (%)]**								0.001
1a		25 (28)	1 (5)	24 (36)				
1b		28 (33)	14 (70)	14 (22)				
2		0 (0)	0 (0)	0 (0)				
3		12 (14)	3 (15)	9 (14)				
4		18 (21)	1 (5)	17 (26)				
Mixed 1a + 1b		3 (4)	1 (5)	2 (3)				
Active and excessive alcohol consumption [n (%)]		9 (11)	1 (5)	8 (12)				0.001
Treatment-experienced (Peg-IFN-based regimens) [n (%)]		31 (36)	4 (20)	27 (41)				0.114
HCV viral load (log UI/l), median (IQR)		6.0 (5.2–6.4)	5.6 (4.9–6.2)	6.1 (5.4 – 6.5)				0.046
AST (IU/l), median (IQR)		48 (30–71)	38 (29–62)	49 (31–74)				0.288
ALT (IU/l), median (IQR)		46 (33–82)	46 (27–91)	46 (34–80)				0.931
Baseline liver stiffness, median (IQR)		11.1 (8.8–20.1)	8.7 (4.7–11.5)	12.7 (9.3–26.3)				0.001
**Baseline fibrosis stage [n (%)]**								0.001
Non-significant fibrosis (F0–F1)		13 (15)	8 (40)	5 (8)				
Significant fibrosis (F2–F3)		41 (48)	8 (40)	33 (50)				
Cirrhosis (F4)		32 (37)	4 (20)	28 (42)				
AST to Platelet Ratio Index (APRI), median (IQR)		0.95 (0.51–1.97)	0.46 (0.39–0.83)	1.11 (0.59–2.76)				0.002
Fibrosis-4 (FIB-4) score, median (IQR)		2.12 (1.54–4.74)	1.56 (1.29–2.00)	2.36 (1.71–5.63)				0.006
Prior liver decompensation [n (%)]		4 (5)	0 (0)	4 (6)				1.000
Platelets/mm^3^ × 1000, median (IQR)	235 (210–262)	157 (102–212)	210 (183–247)	141 (92–186)	< 0.001	0.178	< 0.001	< 0.001
Lymphocites/mm^3^, median (IQR)	2123 (1645–2514)	1908 (1404–2433)	2062 (1523–2261)	1890 (1388–2447)	0.257	0.865	0.212	0.871
CXCL4 (ng/ml), median (IQR)	6425 (4743–9599)	3002 (1435–5894)	2291 (1465–5079)	3174 (1435–6119)	0.002	0.003	0.003	0.649
TGF-β1 (pg/ml), median (IQR)	39,989 (35,356–57,606)	17,797 (11,502–29,674)	20,958 (14,928–37,717)	15,968 (11,068–24,496)	< 0.001	< 0.001	< 0.001	0.079
HGF (pg/ml), median (IQR)	2803 (2294–3111)	4549 (3043–6784)	2738 (1568–4564)	4833 (3488–8394)	< 0.001	0.987	< 0.001	< 0.001
**DAA treatment regimens [n (%)]**								< 0.001
Sofosbuvir/ledipasvir		44 (51)	6 (30)	38 (58)				
Sofosbuvir/simeprevir		2 (2)	0 (0)	2 (3)				
Sofosbuvir/daclastasvir		9 (11)	0 (0)	9 (14)				
Sofosbuvir/velpatasvir		4 (5)	3 (15)	1 (2)				
Ombitasvir/paritaprevir/ritonavir/dasabuvir		21 (25)	7 (35)	14 (22)				
Ombitasvir/paritaprevir/ritonavir		2 (2)	0 (0)	2 (3)				
Elbasvir/grazoprevir		4 (5)	4 (20)	0 (0)				

*HCV* hepatitis C virus, *HIV* human immunodeficiency virus, *IQR* interquartile range, *PWID* people who inject drugs, *AST* aspartate amino-transferase, *ALT* alanine amino-transferase, *CXCL4* chemokine (C-X-C motif) ligand 4, *TGF-β1* transforming growth factor β1, *HGF* hepatocyte growth factor, *DAA* directed antiviral (anti-HCV) agents.

Regarding HIV status, 100% had undetectable HIV viral load (< 50 copies/ml) and the median CD4 + count was 494/ml (312–791), with 9 subjects (14%) presenting severe immunosuppression (< 200 cells/ml).

CXCL4 and TGF-β1 were significantly decreased in HCV-monoinfected and HIV/HCV-coinfected patients in comparison with healthy controls. Serum levels of HGF were significantly increased in HIV/HCV coinfected patients compared with HCV-monoinfected or healthy individuals (Table 1).

### Relationship between liver stiffness and other variables at baseline

LS was directly correlated with serum HGF (r = 0.600, *p* < 0.001) and inversely correlated with TGF-β1 levels (r = −0.374, *p* = 0.001), but not with CXCL4 concentration. TGF-β1 and CXCL4 levels were significantly correlated (r = 0.414, *p* < 0.001). Platelet counts were significantly correlated with serum CXCL4 (r = 0.400, *p* < 0.001) and TGF-β1 levels (r = 0.414, *p* < 0.001). In HIV/HCV coinfected patients, a significant correlation was detected between CD4 + T cell count and CXCL4 concentration (r = 0.309, *p* = 0.015), but not with TGF-β1 or HGF levels.

### Modifications of liver stiffness and serum CXCL4, TGF-β1 and HGF concentrations during follow-up

**Figure 1:**
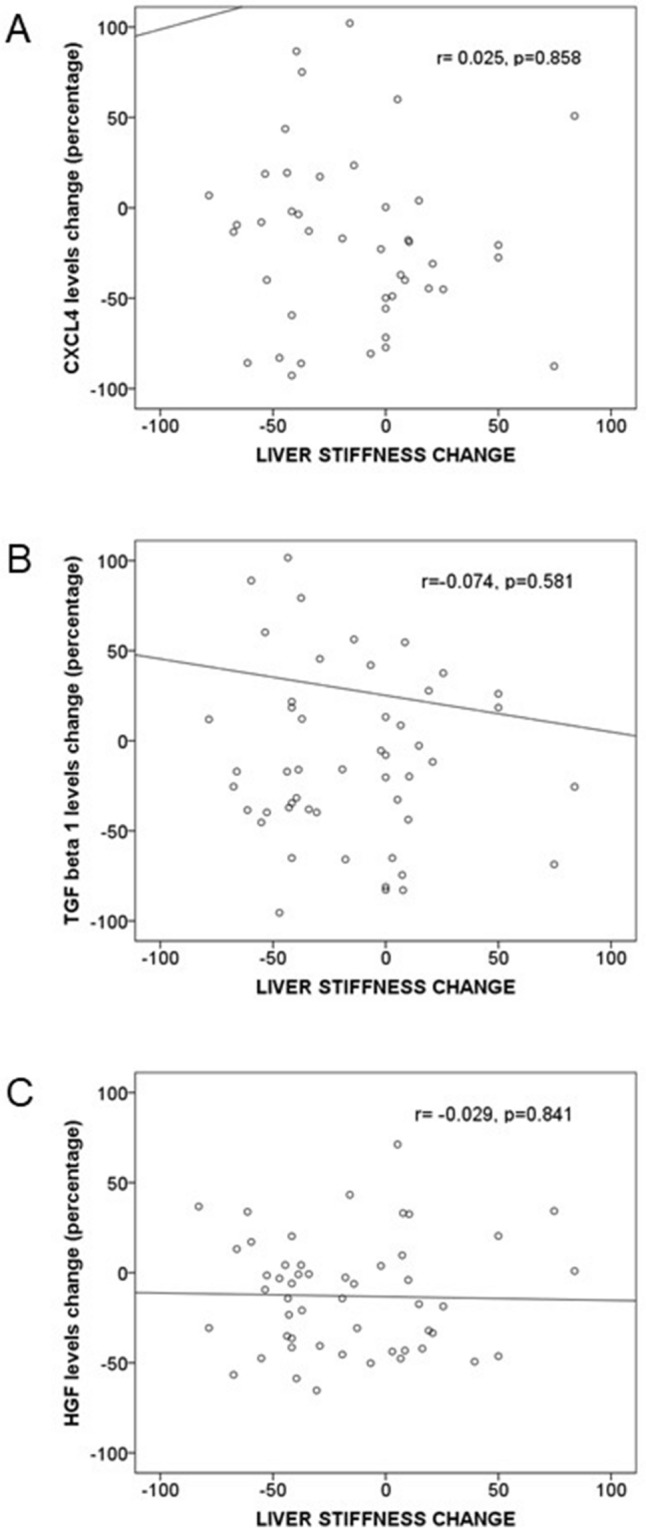
Bivariate correlation between changes in liver stiffness and serum concentrations of chemokine (C-X-C motif) ligand 4 (CXCL4) (**A**); transforming growth factor β1 (TGF-β1) (**B**), and hepatocyte growth factor (HGF) (**C**) concentrations in HCV-infected patients (n = 86), from inclusion (M0) to 12 months after that (M12). Patients started with direct HCV antiviral agents at inclusion and achieved sustained viral HCV response. Data are shown as percentage of change [100 × (M12 − M0)/M0].

**Figure 2:**
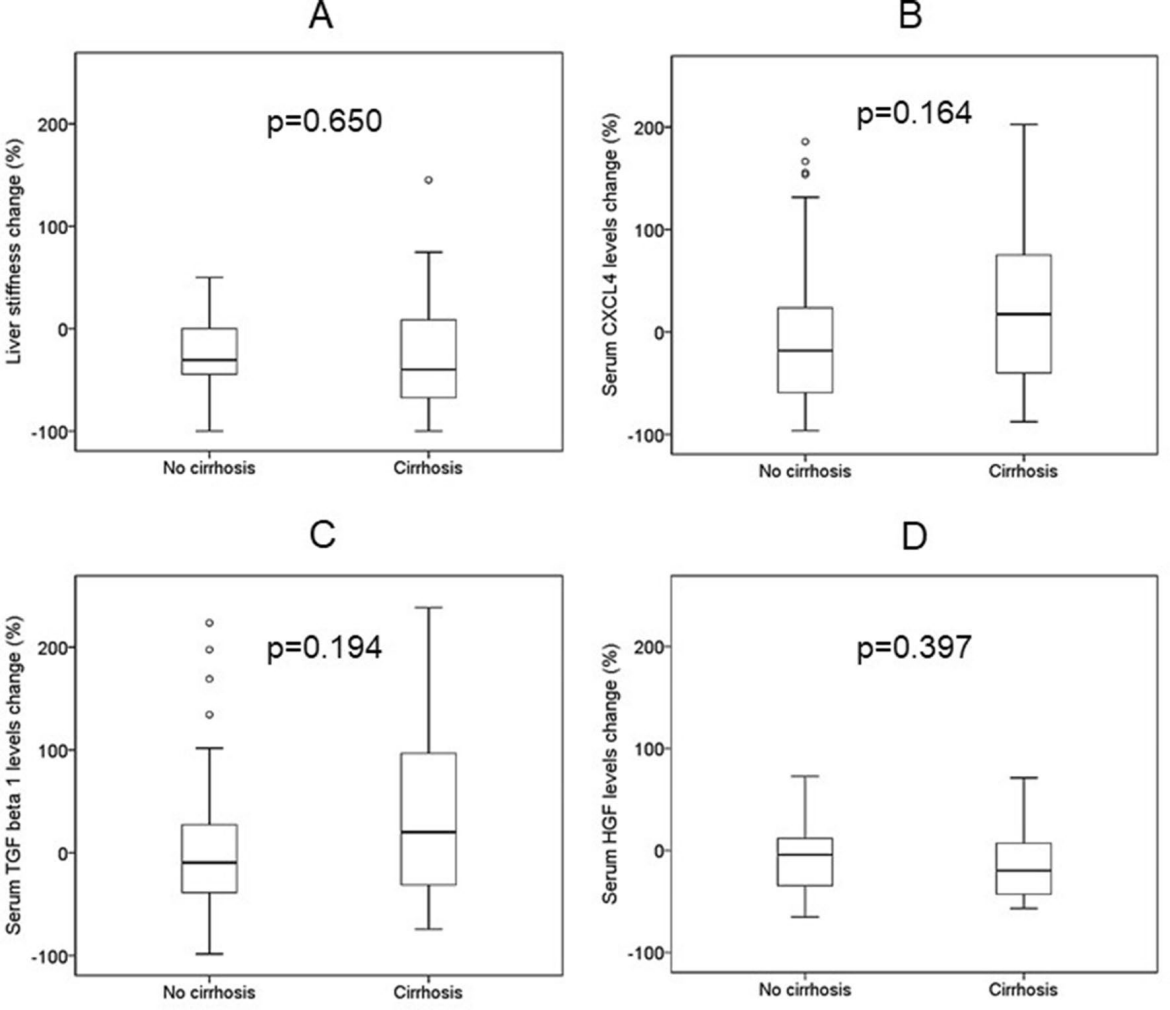
Changes in liver stiffness (**A**), serum chemokine (C-X-C motif) ligand 4 (CXCL4) (**B**), transforming growth factor β1 (TGF-β1) (**C**), and hepatocyte growth factor (HGF) (**D**) concentrations in HCV-infected patients with (n = 32) or without (n = 54) liver cirrhosis, from inclusion to 12 months after that. Patients started with direct HCV antiviral agents at inclusion and achieved sustained viral HCV response. Data are shown as median, interquartile range and range.

Modifications from M0 to M12 in LS, APRI, FIB-4, CXCL4, TGF-β1 and HGF from M0 to M12 are shown in Table 2. A significant decrease of LS, APRI and FIB-4 values were detected from M0 to M12. Likewise, a significant diminution of HGF, but not of CXCL4 or TGF-β1, levels were observed. Changes of LS were not correlated with those of TGF-β1, CXCL4 or HGF levels (Fig. 1). Modifications of LS, APRI and FIB-4 score, CXCL4, TGF-β1 and HGF levels or CXCL4/platelets or TGF-β1/platelets ratios were similar in patients with and without cirrhosis (Fig. 2).

**Table 2:** Modifications of liver stiffness, APRI, FIB-4 score and serum levels of CXCL4, TGF-β1 and HGF levels from baseline (M0) to 12 months after inclusion (M12).

Variable	Baseline values (M0)	Values after 12 months since inclusion (M12)	*p*
Liver stiffness (kPa)	11.1 (8.8–20.1)	9.0 (5.8–18.6)	0.006
AST to platelet ratio Index (APRI), median (IQR)	0.95 (0.51–1.97)	0.37 (0.25–0.67)	< 0.001
Fibrosis-4 (FIB-4) score, median (IQR)	2.12 (1.54–4.74)	1.75 (1.15–2.58)	< 0.001
Serum CXCL4 concentration (ng/ml)	3002 (1435–5894)	2954 (1677–5121)	0.308
Serum TGF-β1 concentration (pg/ml)	17,797 (11,502–29,674)	16,412 (8628–36,031)	0.722
Serum HGF concentration (pg/ml)	4549 (3043–6784)	3803 (2533–5289)	0.001

*HCV* hepatitis C virus, *HIV* human immunodeficiency virus, *IQR* interquartile range, *DAA* directed antiviral (anti-HCV) agents, *CXCL4* chemokine (C-X-C motif) ligand 4, *TGF-β1* transforming growth factor β1, *HGF* hepatocyte growth factor.

**Figure 3:**
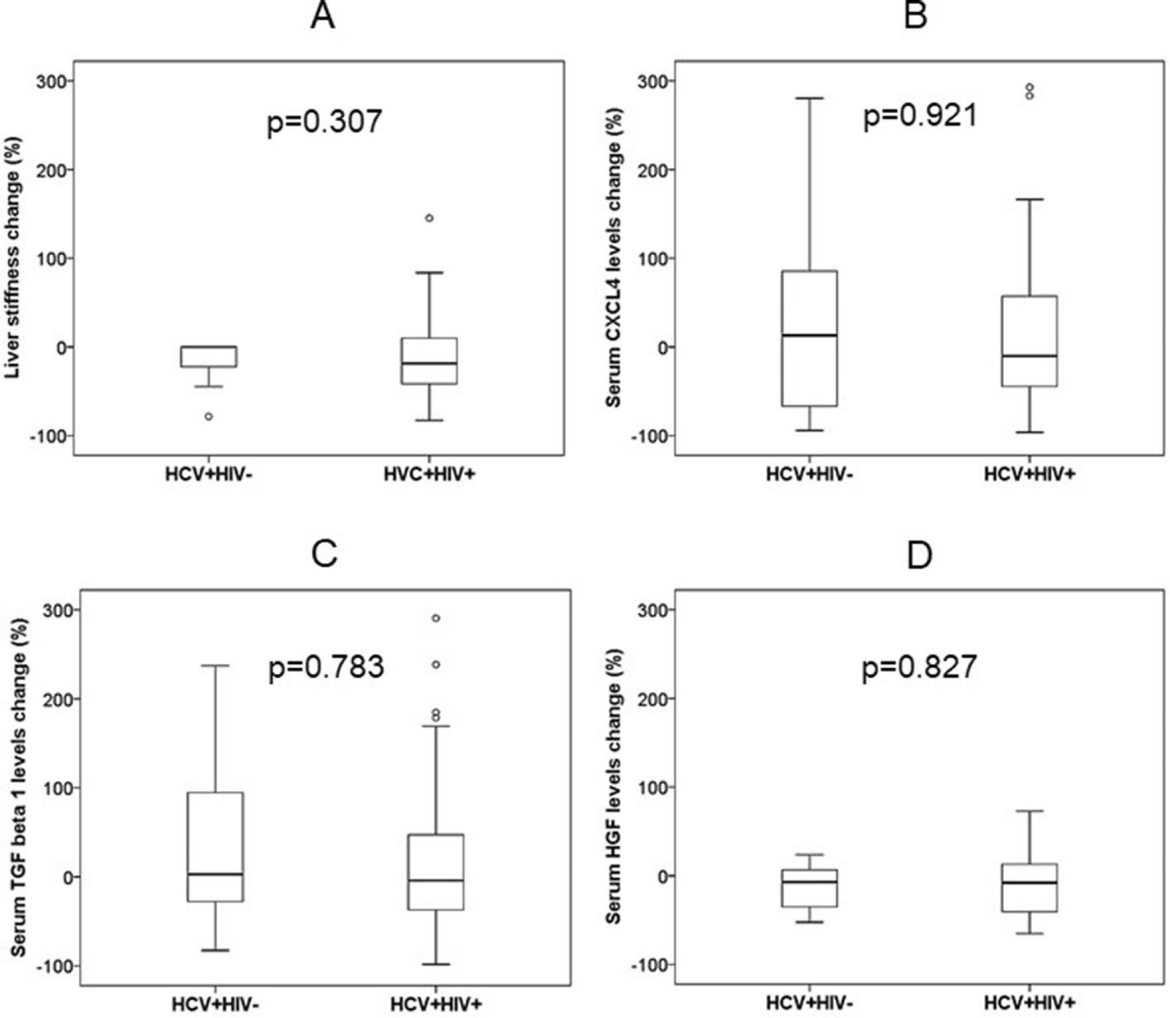
Changes in liver stiffness (**A**), serum chemokine (C-X-C motif) ligand 4 (CXCL4) (**B**), transforming growth factor β1 (TGF-β1) (**C**), and hepatocyte growth factor (HGF) (**D**) concentrations in HCV-infected patients without (HCV + HIV−) (n = 20) or with (HCV + HIV +) (n = 66) HIV coinfection, from inclusion to 12 months after that. Patients started with direct HCV antiviral agents at inclusion and achieved sustained viral HCV response. Data are shown as median, interquartile range and range.

After the follow-up of 12 months, the median of LS decrease was 34% (IQR: − 59, 0). There was no significant difference in the percentage of LS, CXCL4, TGF-β1 and HGF levels decrease among HCV monoinfected and HIV/HCV coinfected patients (Fig. 3).

In HIV/HCV coinfected patients, there was no significant difference in LS change between individuals with a CD4 + T lymphocyte count higher or lower than 200/mm^3^ [− 37 (− 60, + 9) vs − 41 (− 78, + 3), respectively; *p* = 0.770]. Changes of serum TGF-β1, CXCL4 and HGF levels were similar in patients with more or less than 200/mm^3^ (data not shown).

### Modifications of fibrosis stage after SVR

Overall, 26% were reclassified into a different fibrosis stage after follow-up. No change in fibrosis stage was observed in patients with baseline non-significant fibrosis. In those with significant fibrosis (n = 41), 15 of them (37%) were re-classified as non-significant fibrosis and one (2%) as cirrhosis. In those with cirrhosis (n = 32), 7 of them (22%) were re-classified as significant fibrosis.

Because a LS ≤ 9.5 after DAAs is a cutoff indicative for discharge from specialized liver clinic^24^, those patients with a baseline LS > 9.5 (n = 56) were evaluated 12 months after starting DAAs, grouped in function of the LS at M12 (Table 3). 16 of these patients achieved a LS ≤ 9.5 12 months after starting DAAs. After univariate analysis of variables from patients with a baseline LS > 9.5, parameters associated with a LS at M12 ≤ 9.5 were baseline LS, change in the LS after therapy, baseline platelets count and serum HGF levels; baseline TGF-β1 and APRI and risk factor for transmission “people who inject drugs” approached statistical significance. After binary logistic regression, only baseline LS was significantly associated with a M12 LS ≤ 9.5. Range of baseline LS of those with a M12 LS ≤ 9.5 after DAAs was 9.6–21.6, whereas that of patients with a M12 LS > 9.5 was 9.6–53.2.

**Table 3:** Differences of HCV-monoinfected and HIV/HCV coinfected patients with a baseline liver stiffness > 9.5 kPa in function of the achievement of a liver stiffness < 9.5 kPa, measured at 12 months after starting DAAs.

Characteristic	Patients with M12 liver stiffness ≤ 9.5 kPa (n = 16)	Patients with M12 liver stiffness > 9.5 kPa (n = 40)	*p* univariant	Exp (B) (CI 95%)	*p* multivariant
Male sex [n (%)]	11 (69)	34 (85)	0.263		
Age (years), median (IQR)	54 (50–58)	55 (51–60)	0.473		
PWID	4 (25)	22 (55)	0.074		
HIV/HCV coinfection [n (%)]	5 (56)	35 (75)	0.259		
**HCV genotype [n (%)]**			0.368		
1a	6 (38)	12 (30)			
1b	6 (38)	14 (35)			
3	2 (12)	5 (13)			
4	1 (6)	9 (23)			
Mixed 1a + 1b	1 (6)	0 (0)			
Excessive alcohol consumption [n (%)]	1 (6)	3 (7)	0.592		
Treatment-experienced [n (%)]	16 (32)	8 (36)	0.789		
Baseline HCV viral load (log UI/l), median (IQR)	6.3 (4.8–6.8)	5.9 (5.0–6.4)	0.384		
**Baseline fibrosis stage [n (%)]**			0.003		
Significant fibrosis (F2–F3)	12 (75)	12 (30)			
Cirrhosis (F4)	4 (25)	28 (70)			
Baseline liver stiffness, median (IQR)	11.6 (10.3–16.8)	20.1 (13.2–32.9)	0.002	0.887 (0.793–0.992)	0.036
Percentual change of liver stiffness after DAAs (%), median (IQR)	− 46 (− 61, − 41)	5 (− 37, + 18)	< 0.001		
Baseline APRI, median (IQR)	1.00 (0.42–2.06)	1.35 (0.71–2.97)	0.108		
Percentual change of APRI after DAAs (%), median (IQR)	− 44 (− 71, − 38)	− 55 (− 65, − 30)	0.955		
Baseline FIB-4, median (IQR)	1.76 (1.32–4.91)	3.33 (1.80–7.89)	0.054		
Percentual change of FIB-4 after DAAs (%), median (IQR)	− 7 (− 35, + 15)	− 27 (− 44, 0)	0.257		
Platelets/mm^3^ × 1000, median (IQR)	202 (137–232)	114 (78–169)	0.005		
Baseline CXCL4 concentration (ng/ml), median (IQR)	3421 (2109–7199)	2239 (1284–5413)	0.381		
Percentual change of CXCL4 levels after DAAs (%), median (IQR)	7 (− 25, + 75)	1 (− 46, + 89)	0.914		
Baseline TGF-β1 concentration (pg/ml), median (IQR)	20,343 (8599–30,588)	11,778 (7855–22,045)	0.103		
Percentual change of TGF-β1 levels after DAAs (%), median (IQR)	− 2 (− 38, + 59)	− 14 (− 32, + 74)	0.842		
Baseline HGF concentration (pg/ml)	4324 (2960–5686)	6088 (3780–11,031)	0.019		
Percentual change of HGF levels after DAAs (%), median (IQR)	− 5 (− 39, + 8)	− 20 (− 44, + 8)	0.677		

*HCV* hepatitis C virus, *HIV* human immunodeficiency virus, *IQR* interquartile range, *PWID* people who inject drugs, *DAA* directed antiviral (anti-HCV) agents, *APRI* AST to Platelet Ratio Index, *FIB-4* Fibrosis-4 score, *CXCL4* chemokine (C-X-C motif) ligand 4, *TGF-β1* transforming growth factor β1, *HGF* hepatocyte growth factor.

## Discussion

The prospective study presented here showed a significant decline on LS after 12 months with DAA therapy and achieving SVR. The LS improvement was not related with the existence of HIV/HCV coinfection or changes in serum levels of CXCL4, TGF-β1 or HGF.

Besides LS and APRI or FIB-4 score, serum levels of CXCL4, TGF-β1 and HGF were analyzed. It was remarkable that a significant lower concentration of CXCL4 and TGF-β1 was observed in HCV-infected patients compared with healthy controls. On the other hand, LS values were inversely correlated with TGF-β1 in our series, corroborating other authors observations^10–12^. Lower concentrations of fibrosis markers could be explained by one or several of the following: (a) Serum concentrations could be a mirror image of liver levels, justifying the inverse correlation between TGF-β1 and LS. (b) Individuals with severe fibrosis and cirrhosis show decreased serum CXCL4 and TGF-β1 levels owing to reduced numbers of platelets^5^. In fact, platelet counts were significantly correlated with serum CXCL4 and TGF-β1 levels. (c) It could be also possible that the lower concentrations of TGF-β1 and CXCL4 were related with a lower leukocyte or lymphocyte count. Effectively, in HIV/HCV coinfected patients, a significant correlation was detected between CD4 + T cell count (this was not a variable measured in HCV-monoinfected patients) and CXCL4 concentration.

HGF is a mitogenic cytokine implicated in regeneration of hepatic tissue^15^. Because liver cirrhosis is characterized by hepatocyte necrosis, fibrosis and liver regeneration^3^, the direct correlation among serum concentrations of HGF and LS, detected in this and in previous articles^18,29^ was expected: circulating HGF level may reflect hepatic injury during feedback mechanism for repairing the hepatic tissue.

True fibrosis regression (biopsy-proven) is developed when SVR is achieved^30^. Likewise, previous studies have indicated that LS measures may improve following anti-HCV antiviral therapy^31,32^. This was also the case in our series: the median LS decrease after 12 months of follow-up was a 34%. Considering patients with significant fibrosis, a 37% of them achieved posttreatment LS lower than 7.81 kPa (non-significant fibrosis), and a 22% of those with baseline cirrhosis achieved posttreatment LS lower than 15.56 kPa (significant fibrosis, but not cirrhosis).

Some studies have found that TE improvements could be overstated when compared with histologic staging^27,33^, and that this decline might be correlated with inflammation or necrosis improvement^26,34–37^. Our data support this hypothesis: if changes in LS were attributed only to fibrosis modifications, a direct correlation could have been detected with fibrosis marker changes; however, this correlation between changes in serum levels of CXCL4 or TGF-β1 and LS decline was not observed. Furthermore, changes in serum CXCL4 or TGF-β1 concentrations from M0 to M12 were not significant. Attending to these results and measuring liver fibrosis by TE, we can conclude that CXCL4 or TGF-β1 measurements are not convenient for monitoring fibrosis changes.

The technical review on TE by the American Gastroenterological Association suggested that patients without metabolic comorbidities, history of alcohol excess, or HBV–HIV coinfection, and with a liver stiffness of ≤ 9.5 kPa after sustained HCV viral response may be considered for discharge from a specialized liver clinic^24^. A M12 LS ≤ 9.5 kPa was achieved by a 29% (16 out of 56) of those with baseline LS higher than this cutoff point. Baseline LS, change in the LS after therapy, baseline platelets count and serum HGF levels (but not CXCL4 or TGF-β1 concentration) were associated with the achievement of a M12 LS ≤ 9.5 kPa in the univariate analysis.

Baseline LS, but not baseline APRI or FIB-4, was able to discriminate between patients with an initial LS > 9.5 who achieved a final LS lower than the mentioned cut-off point. In fact, only the baseline LS was independently associated with a M12 LS ≤ 9.5. However, patients with a wide range of baseline LS (9.6–21.6 kPa) could achieve that cutoff point, limiting the usefulness of baseline LS as predictive marker.

It has been stated that the achievement of SVR plays a determinant role in preventing additional liver damage^32^. This is even more remarkable in HIV/HCV coinfected patients^33^. Thus, a particular analysis of these patients was performed. They were characterized by a relatively preserved immune status, as proved by a median CD4 + count of 494/mm^3^, and undetectable HIV viral load. The LS and the proportion of them with liver cirrhosis was significantly higher than those of HCV-monoinfected patients. After 12 months of follow-up and having achieved SVR, HIV/HCV coinfected patients showed similar change of LS to HCV-monoinfected individuals. This decrease was similar in patients with more or less than 200 CD4 + lymphocytes/mm^3^, although the number of patients with less than 200/mm^3^ (n = 9) limits the extrapolation of results. Serum levels of profibrotic (TGF-β1 and CXCL4) markers and changes of these serum concentrations were similar in HCV-monoinfected and HIV/HCV coinfected individuals, suggesting that liver fibrosis regulatory mechanisms function in a similar mode in both groups of patients. Attending to direct correlation among serum concentrations of HGF and LS, serum HGF were significantly increased in HIV/HCV coinfected patients, in which the proportion of cirrhotic patients was higher. However, modification of serum HGF from M0 to M12 was similar in HCV-monoinfected and HIV/HCV coinfected individuals.

Our study might have some limitations. First, the sample size limited our ability to control for confounding factors that might interfere with SVR; the power of the study was insufficient to identify differences in several covariates, such as genotype, immune status or HCV viral load. Second, histologic assessment was not available, so correlations between TE results and biopsies could not be performed. Third, as it has been previously detected in articles that have compared HIV/HCV coinfected and HCV-monoinfected patients^38,39^, HIV/HCV coinfected individuals are younger, with a predominance of drug use as risk factor. HCV genotype 1b predominantly affects to HCV-monoinfected individuals. However, these factors were not significantly correlated with serum levels of CXCL4, TGF-β1 and HGF or with LS measures or associated with an achievement of a LS < 9.5 kPa in the bivariate or multivariate analyses.

In summary, a third of HCV-infected patients that achieved HCV eradication experienced a significant reduction of LS, assessed by TE. LS decline was similar in HCV-monoinfected and HIV/HCV coinfected patients. The significance of this reduction is unclear, because there was no correlation with change in serum levels of fibrosis markers CXCL4 and TGF-β1 or in the proliferative HGF.

## Methods

### Patients and study design

This is a prospective cohort study of HCV-infected patients conducted at the Hospital Universitario Puerta del Mar (Cádiz, Spain). Two groups of patients were analyzed: HCV monoinfected patients (n = 20) and HIV/HCV coinfected patients (n = 66). A group of healthy individuals (n = 15) served as controls.

Subjects were eligible for analysis if they were treated with DAAs; started the therapy between 1 January 2016 and 31 December 2018, and achieved sustained virological response. They underwent a baseline transient elastography (TE) and were taken a serum sample at inclusion and at least another TE and serum sample 12 months after starting anti-HCV treatment.

All patients with detectable serum HCV RNA naïve to DAAs were consecutively enrolled. Anti-HCV therapy was performed under routine clinical care conditions, according to the best medical judgment and the prevailing HCV treatment guidelines at the time [www.seimc.org], based on HCV genotype, history of HCV treatment, drug interactions with antiretroviral treatment (ART) against HIV, and liver fibrosis assessed through TE. Anti-HCV therapy was administered for 12 weeks in all cases. In HIV/HCV coinfected patients, there were no modifications in the ART during the study period.

The exclusion criteria were hepatitis B virus coinfection, other concomitant causes of liver disease, including active alcohol abuse, active infections, and past or present treatment with steroids or immunosuppressive drugs prior to starting anti-HCV therapy.

The follow-up period covered from the date of the treatment onset (M0) until 12 months (M12) after starting anti-HCV treatment. Information on blood cells counts, kidney and liver function parameters, and virological markers was collected at M0, at week 12 posttreatment visits and at M12. Data about LS and fibrosis (CXCL4 and TGF-β1)- or proliferation (HGF)-related variables was collected at M0 and M12.

### Liver fibrosis staging

LS was examined through TE by the FibroScan system (Echosens, Paris, France) at M0 and M12. A minimum of 10 LS measurements were required. The median value was assumed to be representative of LS. A set of measurements was considered to be reliable if the success rate was ≥ 60% and the interquartile range was less than one-third of the median LS value. Unreliable measurements of LS were excluded. All measurements were obtained by two trained operators (MRP and JAGG) using a single device. Results, expressed in kilopascals (kPa), were categorized in three stages as follows: non-significant fibrosis (F0–F1), under 7.81 kPa; significant fibrosis (F2–F3), equal or more than 7.81 kPa; cirrhosis (F4), ≥ 15.56 kPa, according to Stebbing’s meta-analysis^40^. Besides fibrosis, other factors may also result in increased liver stiffness, such as the presence of severe hepatic inflammation, extrahepatic arteriovenous or biliary obstruction, and congestive heart failure^24^. They were excluded by clinical and complementary explorations.

AST to platelets ratio index (APRI)^20^ and Fibrosis-4 (FIB-4) score^21^ were also calculated.

### Variables

Change in LS was the dependent variable and was defined by the difference between the values obtained at M12 and the M0 by TE, measured in kPa, divided by the M0 value [(M12 liver stiffness − M0 liver stiffness)/M0 liver stiffness].

Independent variables included: demographic factors [sex, age, HIV/HCV transmission mechanism, alcohol consumption (> 50 g/day, for more than 5 years)]; HIV-coinfection and HIV-related factors (viral load, CD4 + count and percentage); HCV-related factors (viral load, genotype, previous treatment experience); (d) other laboratory data (serum transaminases); (e) treatment-related factors (DAA regimen); (f) serum levels of fibrosis (CXCL4 and TGF-β1) and proliferation (HGF)-related markers; (g) APRI and FIB-4 score.

Changes from M0 to M12 of LS, APRI, FIB-4, or serum fibrosis and proliferation markers were calculated as the values at M12 minus the values at M0, divided by the values at M0 [(M12 value − M0 value)/M0 value].

SVR at week 12 (SVR12) was defined as HCV RNA undetectable at week 12 after the treatment had ended.

### Statistical analysis

For comparisons of patient’s characteristics, we used the Fisher and Chi-square tests (categorical variables) or the Student’s t test and Mann–Whitney test (continuous variables), depending on the normality of distributions.

The primary outcome was the assessment of evolution of LS after 12 months of follow-up. Likewise, a LS equal or lower of 9.5 was selected, because a post-SVR LS of ≤ 9.5 kPa may be considered as a cutoff point for discharge from a dedicated liver clinic^24^. To establish nonadjusted associations between LS decline and patient’s clinical or analytical findings, we used univariant models. Multivariable linear regression models were implemented to analyze the impact on LS of statistical significant independent variables. The study length was calculated from the date of the DAA regimen prescription to the 12 months after starting anti-HCV therapy. Statistical analyses were performed with SPSS for Windows version 22.0 (SPSS, Inc), considering a value of *p* < 0.05 significant.

### Ethical aspects

The study was designed and conducted following the Helsinki declaration. The Ethics Committee of the Hospital Universitario Puerta del Mar (Cádiz) approved the study, and all the patients gave their written informed consent.
